# Event-Based Face Detection and Tracking Using the Dynamics of Eye Blinks

**DOI:** 10.3389/fnins.2020.00587

**Published:** 2020-07-27

**Authors:** Gregor Lenz, Sio-Hoi Ieng, Ryad Benosman

**Affiliations:** ^1^INSERM UMRI S 968, Sorbonne Université, UPMC Univ. Paris, UMRS 968, Paris, France; ^2^CNRS, UMR 7210, Institut de la Vision, Paris, France; ^3^Departments of Ophthalmology/ECE/BioE, University of Pittsburgh, Pittsburgh, PA, United States; ^4^Robotics Institute, Carnegie Mellon University, Pittsburgh, PA, United States

**Keywords:** face detection, face tracking, event-based computation, neuromorphic vision, silicon retina

## Abstract

We present the first purely event-based method for face detection using the high temporal resolution properties of an event-based camera to detect the presence of a face in a scene using eye blinks. Eye blinks are a unique and stable natural dynamic temporal signature of human faces across population that can be fully captured by event-based sensors. We show that eye blinks have a unique temporal signature over time that can be easily detected by correlating the acquired local activity with a generic temporal model of eye blinks that has been generated from a wide population of users. In a second stage once a face has been located it becomes possible to apply a probabilistic framework to track its spatial location for each incoming event while using eye blinks to correct for drift and tracking errors. Results are shown for several indoor and outdoor experiments. We also release an annotated data set that can be used for future work on the topic.

## 1. Introduction

This paper introduces an event-based method to detect and track faces from the output of an event-based camera. We also release a dataset of 50 recordings, consisting of a mix of indoor and outdoor conditions. 25 of those recordings have been annotated for a total of 265 blinks^1^. The method exploits the dynamic properties of human faces to detect, track and update multiple faces in an unknown scene. Although face detection and tracking are considered practically solved in classic computer vision, it is important to emphasize that current performances of conventional frame based techniques come at a high operating computational cost after days of training on large databases of static images. Event-based cameras record changes in illumination at high temporal resolutions (in the range of 1μ*s* to 1 ms) and are therefore able to acquire the dynamics of moving targets present in a scene (Lichtsteiner et al., 2008). In this work we will rely on eye blink detection to determine the presence of a face in a scene to in a second stage initialize the position of a bayesian tracker. The permanent computation of eye blinks will allow to correct tracking drifts and reduce localization errors over time. Blinks produce a unique space-time signature that is temporally stable across populations and can be reliably used to detect the position of eyes in an unknown scene. This paper extends the sate-of-art by:

- Implementing a low-power human eye-blink detection that exploits the high temporal precision provided by event-based cameras.
- Tracking of multiple faces simultaneously at μs precision, once they have been detected.

The developed methodology is entirely event-based as every event output by the camera is processed into an incremental, non-redundant scheme rather than creating frames from events to recycle existing image-based methodology. We also show that the method is inherently robust to scale changes of faces by continuously inferring the scale from the distance of the two eyes of the tracked face from detected eye blinks. The method is compared to existing image-based face detection techniques (Viola and Jones, 2004; Liu et al., 2016; Jiang and Learned-Miller, 2017; Li and Shi, 2019). It is also tested on a range of scenarios to show its robustness in different conditions: indoors and outdoors scenes to test for the change in lighting conditions; a scenario with a face moving close and moving away to test for the change of scale, a setup of varying pose and finally a scenario where multiple faces are detected and tracked simultaneously. Comparisons with existing frame-based methods are also provided.

### 1.1. Event-Based Cameras

Biomimetic event-driven time-based vision sensors are a novel class of vision device that—like the biological retina—are driven by “events”happening within the visual scene. They are not like conventional vision sensors, which are driven by artificially created timing and control signals (e.g., frame clock) that have no relation whatsoever to the source of the visual information (Lichtsteiner et al., 2008).

Over the past few years, a variety of these event-based devices has been developed, including temporal contrast vision sensors that are sensitive to relative luminance change (Lichtsteiner et al., 2008), some also providing also absolute light measurement (Posch et al., 2011).

**Figure 1:**
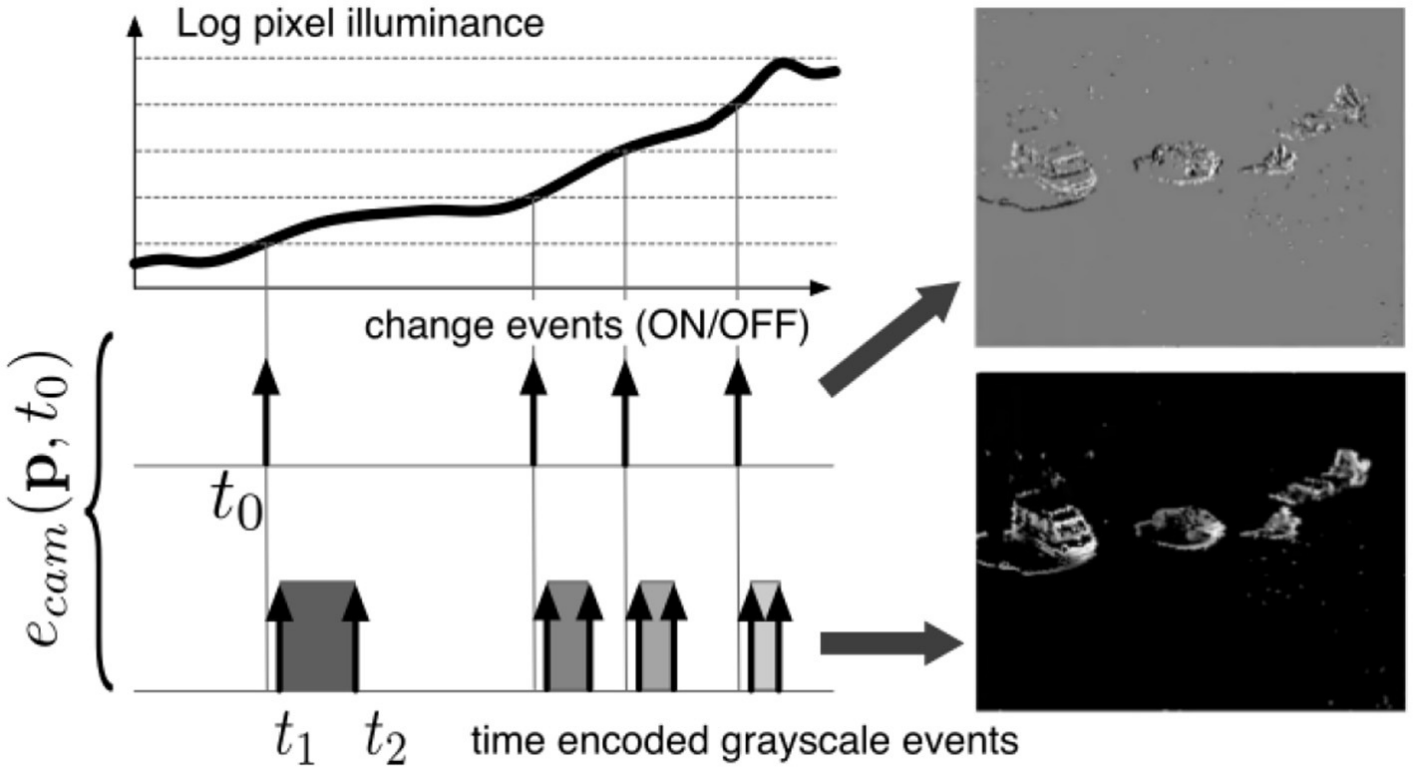
Working principle of the event-based camera and two types of events. (1) Change event of type ON is generated at *t*_0_ as voltage generated by incoming light crosses a voltage threshold. (2) Time *t*_2_ − *t*_1_ to receive a certain amount of light is converted into an absolute gray-level value, emitted at *t*_2_ used for ground truth in the paper.

These vision sensors output visual information about the scene in the form of asynchronous address events using the Address Event Representation protocol and encode the visual information in the time dimension rather than as a voltage, charge, or current. The novel algorithm for face detection and tracking we propose in this paper is designed to take advantage of the high temporal resolution data representation provided by event-based cameras. The operating principle of these sensor is shown in Figure 1. An event is defined as the n-tuple: *ev* = (*x, y, t, p*), where (*x, y*) are the pixel coordinates, *t* the time of occurrence and *p* is the polarity. Variations of event-based cameras implement additional functionality.

In this work, we are using the Asynchronous Time-based Image Sensor (ATIS) (Posch et al., 2011) as it also provides events that encode absolute luminance information. This additional information allows for direct and easier comparisons with the frame-based world.

### 1.2. Face Detection

State-of-the-art face detection relies on neural networks that are trained on large databases of face images, to cite the latest from a wide literature, readers should refer to Yang et al. (2017), Jiang and Learned-Miller (2017), and Sun et al. (2018). Neural Networks usually rely on intensive computation that necessitates dedicated hardware co-processors (usually GPU) to enable real-time operations (Ren et al., 2008). Currently dedicated chips such as Google's Tensor Processing Unit or Apple's Neural Engine have become an essential tool for frame-based vision. They are designed to accelerate matrix multiplications at the core of neural networks inference. However, the computation costs associated to these computations are extremely high (thousands of Watts).

Dedicated blink detection using conventional frame-based techniques operate on a single frame. To constrain the region of interest, a face detection algorithm is used beforehand (Noman and Ahad, 2018). In an event-based approach, the computational scheme can be inverted as detecting blinks is the mechanism that drives face detection.

### 1.3. Human Eye Blinks

Humans blink synchronously in correlation to their cognitive activities and more often than required to keep the surface of the eye hydrated and lubricated. Neuroscience research suggests that blinks are actively involved in the release of attention (Nakano et al., 2013). Generally, the observed eye blinking rates in adults depend on the subject's activity and level of focus. Rates can range from 3*blinks*/*min* when reading to up to 30*blinks*/*min* during conversation (Table 1). Fatigue significantly influences blinking behaviors, increasing both rate and duration (Stern et al., 1994). In this paper we will not consider these boundary cases that will the be subject of further work on non-intrusive driver monitoring (Häkkänen et al., 1999; Wang et al., 2006). A typical blink duration is between 100 and 150*ms* (Benedetto et al., 2011). It shortens with increasing physical workload, increased focus or airborne particles related to air pollution (Walker et al., 2001).

**Table 1:** Mean blinking rates according to Bentivoglio et al. (1997) and Stern et al. (1994).

**Activity (blinks/min)**	**Bentivoglio et al. (1997)**	**Stern et al. (1994)**
Reading	4.5	3–7
At rest	17	–
Communicating	26	–
Not reading	–	15–30

**Figure 2:**
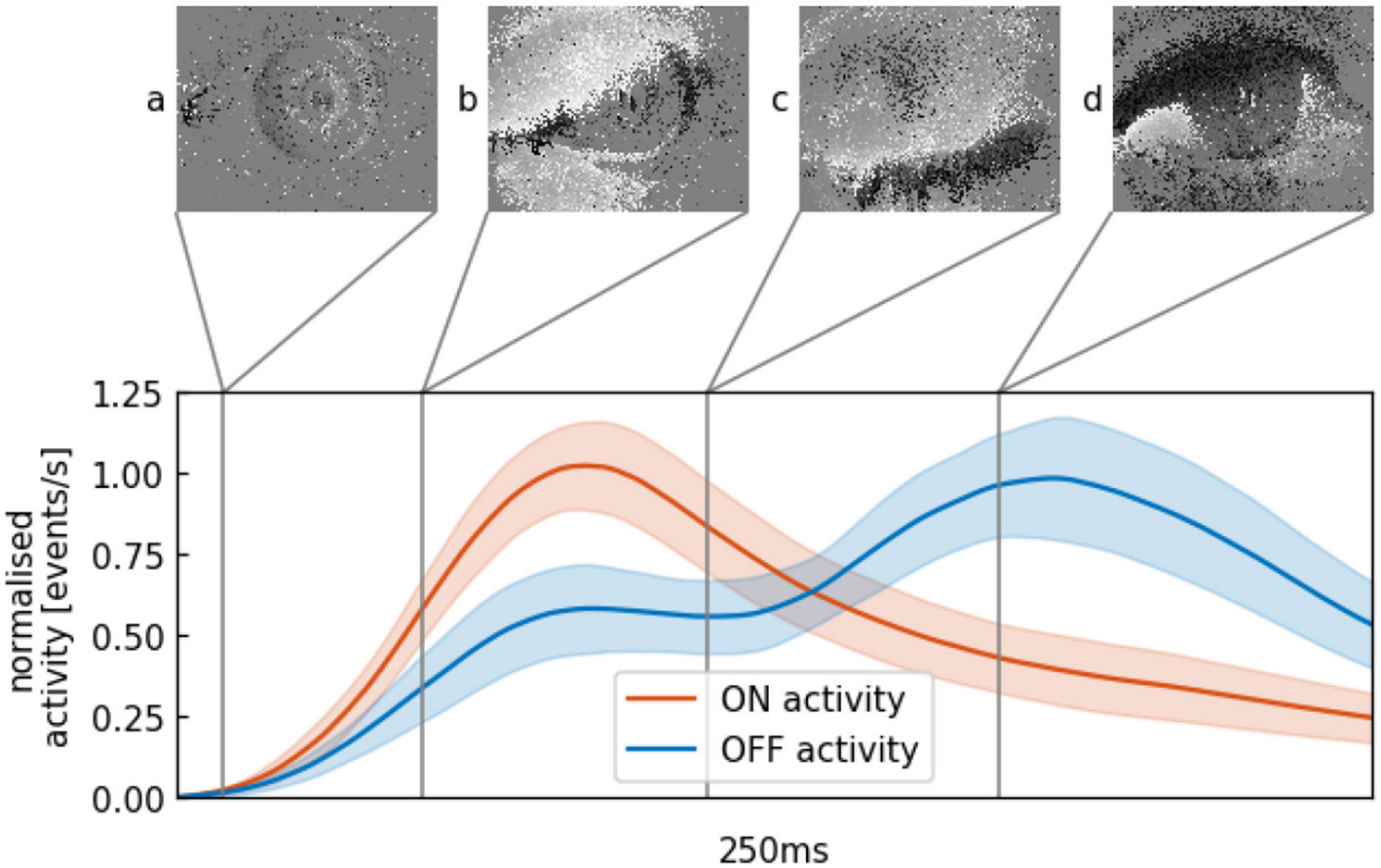
Mean and variance of the continuous activity profile of averaged blinks in the outdoor data set with a decay constant of 50 ms. **(A)** Minimal movement of the pupil, almost no change is recorded. **(B)** Eye lid is closing within 100 ms, lots of ON-events (in white) are generated. **(C)** Eye is in a closed state and a minimum of events is generated. **(D)** Opening of the eye lid is accompanied by the generation of mainly OFF-events (in black).

To illustrate what happens during an event-based recording of an eye blink, Figure 2 shows different stages of the eye lid closure and opening. If the eye is in a static state, few events will be generated (Figure 2A). The closure of the eye lid happens within 100 ms and generates a substantial amount of ON events, followed by a slower opening of the eye (Figures 2C,D) and the generation of primarily OFF events. From this observation, we devise a method to build a temporal signature of a blink. This signature can then be used to signal the presence of a single eye or pair of eyes in the field of view that can then be interpreted as the presence of a face.

## 2. Methods

### 2.1. Temporal Signature of an Eye Blink

**Figure 3:**
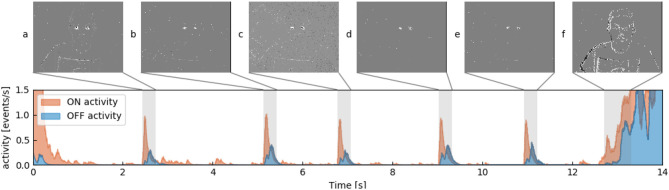
Showing ON (red) and OFF (blue) activity for one tile which lines up with one of the subject's eyes. Multiple snapshots of accumulated events for 250 ms are shown, which corresponds to the gray areas. **(A–E)** Blinks. Subject is blinking. **(F)** Subject moves as a whole and a relatively high number of events is generated.

Eye blinks can be represented as a temporal signature. To build a canonical eye blink signature *A*(*t*_*i*_) of a blink, we convert events acquired from the sensor into temporal activity. For each incoming event *ev* = (*x*_*i*_, *y*_*i*_, *t*_*i*_, *p*_*i*_), we update *A*(*t*_*i*_) as follows:

(1) $$\begin{array}{l} A\left(t_{i}\right)=\{\begin{array}{cc} A_{ON}\left(t_{i}\right)=A_{ON}\left(t_{u}\right)e^{-\frac{t_{i}-t_{u}}{\tau }}+\frac{1}{scale} & \text{if }p_{i}\text{=ON}\\ A_{OFF}\left(t_{i}\right)=A_{OFF}\left(t_{v}\right)e^{-\frac{t_{i}-t_{v}}{\tau }}+\frac{1}{scale} & \text{if }p_{i}\text{=OFF} \end{array} \end{array}$$

where *t*_*u*_ and *t*_*v*_ are the times an ON or OFF event occurred before *t*_*i*_. The respective activity function is increased by $\frac{1}{scale}$ each time *t*_*n*_ an event ON or OFF is registered (light increasing or decreasing). The quantity *scale* initialized to 1 acts as a corrective factor to account for a possible change in scale, as a face that is closer to the camera will inevitably trigger more events. Figure 3 shows the two activity profiles where 5 profiles of a subject's blinks are shown, as well as much higher activities at the beginning and the end of the sequence when the subject moves as a whole. From a set of manually annotated blinks we build such an activity model function as shown in Figure 2 where red and blue curve respectively represent the ON and OFF parts of the profile.

Our algorithm detects blinks by checking whether the combination of local ON- and OFF-activities correlates with the canonical model of a blink that had previously been “learned” from annotated material. To compute the local activity, the overall input focal plane is divided into one grid of *n* × *n* tiles, overlapped with a second similar grid made of (*n* − 1) × (*n* − 1) tiles. Each of these are rectangular patches, given the event-camera's resolution of 304 × 240 pixels. The second grid is shifted by half the tile width and height to allow for redundant coverage of the focal plane. In this work we set experimentally *n* = 16 as it corresponds to the best compromise between performance and the available computational power of the used hardware.

#### 2.1.1. Blink Model Generation

A total of *M* = 120 blinks from six subjects are manually annotated from the acquired data to build the generic model of an eye blink shown in Figure 2. Each blink, extracted within a time window of 250 ms is used to compute an activity function as defined in Equation (1). The blink model is then obtained as the average of these activity functions:

(2) $$\begin{array}{l} B\left(t\right)=\{\begin{array}{ll} B_{ON}\left(t\right)=\overset{M}{\underset{k=1}{\sum }}\frac{A_{ON}\left(t\right)}{M}, & \text{if }p_{i}\text{=ON}\\ B_{OFF}\left(t\right)=\overset{M}{\underset{k=1}{\sum }}\frac{A_{OFF}\left(t\right)}{M}, & \text{if }p_{i}\text{=OFF} \end{array} \end{array}$$

To provide some robustness and invariance to scale and time changes to the blink model, we also define N, the number of events per unit of time and normalized by the scale factor. This number provides the number of samples necessary to calculate the cross-correlation to detect blink as explained in section 2.1.2. Formally, $N=\left\lfloor \frac{\#\text{events}}{T.scale}\right\rfloor$, where ⌊.⌋ is the floor function giving the largest integer smaller than $\frac{\#\text{events}}{T.scale}$.

Finally, we used two different models for indoor and outdoor scenes, as experiments showed that the ratio between ON and OFF events change substantially according to the lighting conditions. Although the camera is supposed to be invariant to absolute illumination, this is practically not the case due to hardware limitations of early camera generation that we used for this paper.

#### 2.1.2. Sparse Cross-Correlation

When streaming data from the camera, the most recent activity within a *T* = 250 ms time window is taken into account in each tile to calculate the similarity score, here the cross-correlation score, for the ON and OFF activities. This cross-correlation is only computed if the number of recent events exceeds an amount *N* defined experimentally according to the hardware used. The cross-correlation score between the incoming stream of events and the model is given by:

(3) $$\begin{array}{l} C\left(t_{k}\right)=\alpha C_{ON}\left(t_{k}\right)+\left(1-\alpha \right)C_{OFF}\left(t_{k}\right), \end{array}$$

where

(4) $$\begin{array}{l} C_{p}\left(t_{k}\right)=\overset{N}{\underset{i=0}{\sum }}A_{p}\left(t_{i}\right)B_{p}\left(t_{i}-t_{k}\right), \end{array}$$

with *p*∈{*ON, OFF*}. The ON and OFF parts of the correlation score are weighted by a parameter α set experimentally that tunes the contribution of the ON vs OFF events. This is a necessary step -as explained in the previous section-, due to the camera manual parameter settings, the amount of ON and OFF events are usually not balanced. For all experiments, α is set to $\frac{2}{3}$.

**Figure 4:**
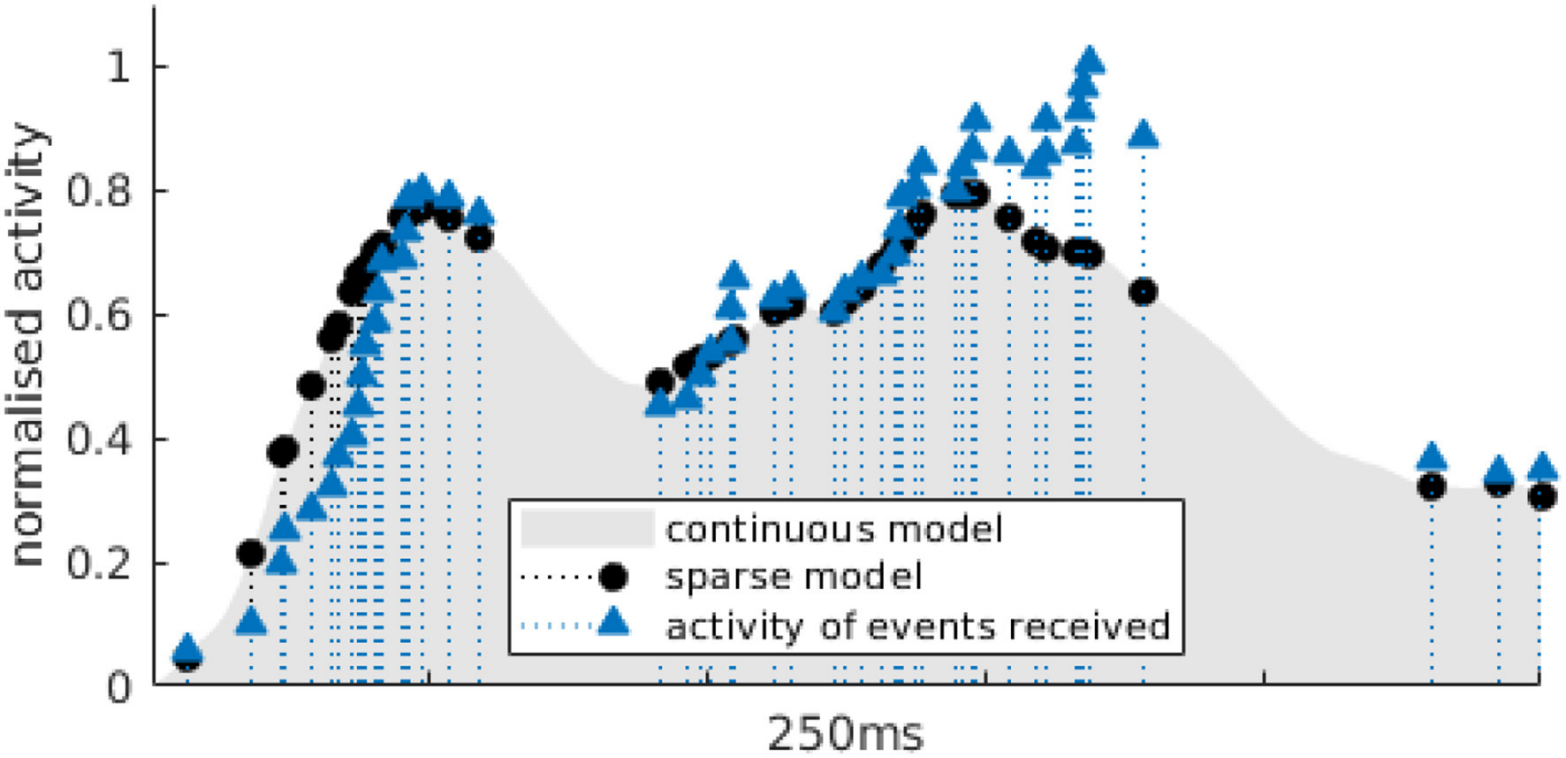
Example of the samples used to calculate the sparse cross-correlation for the OFF activity of an actual blink. The gray area represents *B*_*OFF*_, the activity model for OFF events (in that particular example, it is previously built for outdoor data sets). Blue triangles correspond to the activity *A*(*t*_*i*_) for which events have been received in the current time window. Black dots are the *B*_*OFF*_(*t*_*i*_), the value of activity in the model at the same times-tamps as incoming events.

It is important for implementation reasons to compute the correlation as it appears in Equation (4). While it is possible to calculate the value of the model *B*(*t*−*t*_*k*_) at anytime *t*, samples for *A* are only known for the set of times {*t*_*i*_}, from the events. This is illustrated as an example by Figure 4, for an arbitrary time *t*_*k*_, where triangles outline the samples of the activity for calculated events at *t*_*i*_ and the circles show the samples calculated at the same time *t*_*i*_ with the model. If *C*(*t*_*i*_) exceeds a certain threshold, we create what we call a blink candidate event for the tile in which the event that triggered the correlation occurred. Such a candidate is represented as the n-tuple *eb* = (*r, c, t*), where (*r, c*) are the row and column coordinates of the grid tile and *t* is the timestamp. We do this since we correlate activity for tiles individually and only in a next step combine possible candidates to a blink.

#### 2.1.3. Blink Detection

To detect the synchronous blinks generated by two eyes, blink candidates across grids generated by the cross-correlation are tested against additional constraints for verification. As a human blink has certain physiological constraints in terms of timing, we check for temporal and spatial coherence of candidates in order to find true positives. The maximum temporal difference between candidates will be denoted as Δ*T*_*max*_ and is set experimentally to 50 ms, the maximum horizontal spatial disparity Δ*H*_*max*_ is set to half the sensor width and the maximum vertical difference Δ*V*_*max*_ is set to a fifth of the sensor height. Following these constraints we will not detect blinks that happen extremely close to the camera or stem from substantially rotated faces. Algorithm 1 summarizes the set of constraints to validate the detection of a blink. The scale factor here refers to a face that has already been detected.

**Algorithm 1:**
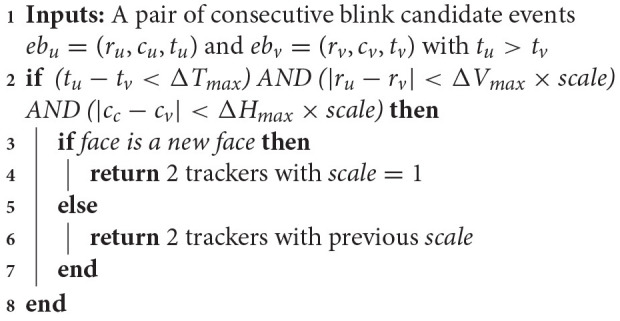
Blink detection

### 2.2. Gaussian Tracker

Once a blink is detected with sufficient confidence, a tracker is initiated at the detected location. We use trackers such as the ones presented in Lagorce et al. (2015) that rely on bivariate normal distributions to locally model the spatial distribution of events. For each event, every tracker is assigned a score that represents the probability of the event to belong to the tracker:

(5) $$\begin{array}{l} p\left(u\right)=\frac{1}{2\pi }|\Sigma {|}^{-\frac{1}{2}}e^{-\frac{1}{2}{\left(\text{u}-\mu \right)}^{T}\Sigma^{-1}\left(\text{u}-\mu \right)} \end{array}$$

where *u* = [*x, y*]^*T*^ is the pixel location of the event, is covariance matrix Σ that defines the shape and size of the tracker. The tracker with the highest probability is updated according to the activity of pixels and also according to the estimated distance between the spatial locations of the detected eyes.

### 2.3. Global Algorithm

The detection and tracking blocks combined operations are summarized by following algorithm:

**Algorithm 2:**
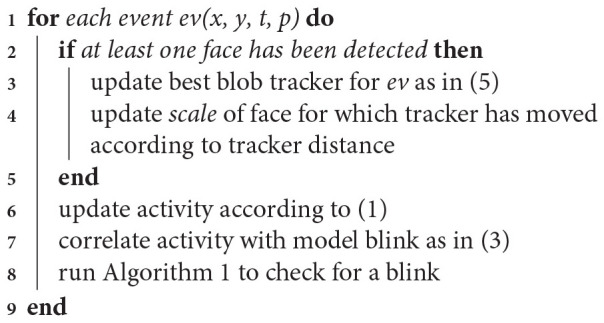
Event-based face detection and tracking algorithm

## 3. Experiments and Results

We evaluated the algorithm's performance by running cross-validation on a total of 48 recordings from 10 different subjects, comprising 248 blinks. The recordings are divided into five sets of experiments to assess the method's performances under realistic constraints encountered in natural scenarios. The event-based camera is set static. We test the following scenarios of sequences of:

- indoor and outdoor sequences showing a single subject moving in front of the camera,
- a single face moving back and forth w.r.t. the camera to test the robustness of scale change,
- several subjects discussing, facing the camera to test for multi-detection,
- a single face changing its orientation w.r.t. the camera to test for occlusion resilience.

The presented algorithm has been implemented in C++ and runs in real-time on an Intel Core i5-7200U laptop CPU. We are quantitatively assessing the proposed method's accuracy by comparing it with state of the art and gold standard face detection algorithms from frame-based computer-vision. As these approaches require frames, we are generating gray-levels from the camera when this mode is available. The Viola and Jones (2004) algorithm (VJ) provides the gold standard face detector but we also considered the Faster R-CNN (FRCNN) from Ren et al. (2015) and the Single Shot Detector (SSD) network from Liu et al. (2016) that have been trained on the Wider Face (Yang et al., 2016) data set. This allows us to compare the performances of the event-based blink detection and tracking with state-of-the-art face detectors based on deep learning. Finally, we also tested a conventional approach that combines CNN and correlation filter presented in Li and Shi (2019). It is referred to as the “Correlation Filter” (CF) for the rest of the paper. This technique, however, relies on creating frames by summing the activities of pixels within a predefined time window.

An important statement to keep in mind is that the proposed blink detection and face tracking technique requires reliable detection. We do not actually need to detect all blinks since a single detection is already sufficient to initiate the trackers. Additional blink detections are used to correct a trackers' potential drifts regularly.

### 3.1. Indoor and Outdoor Face Detection

**Figure 5:**
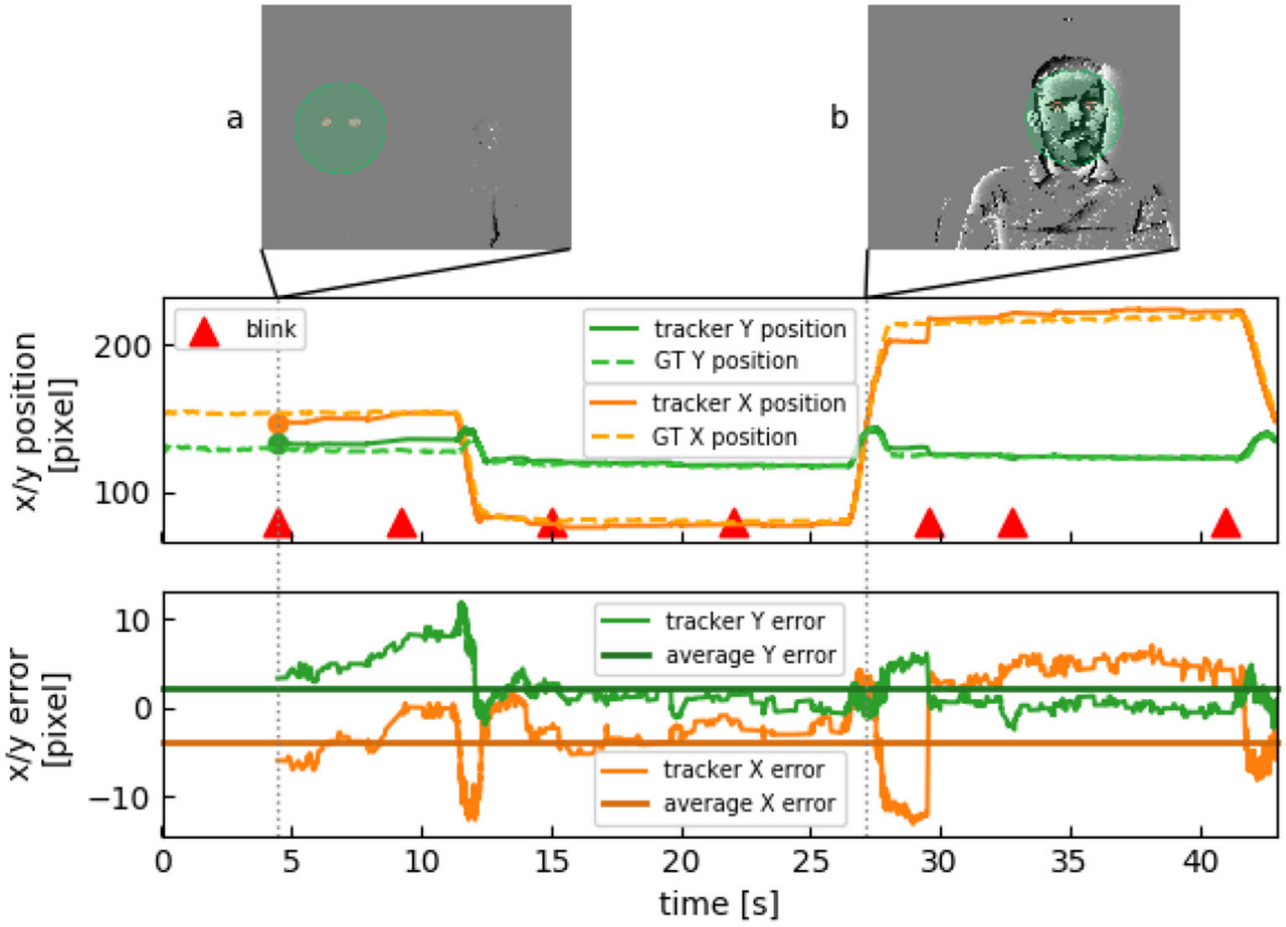
Face tracking of one subject over 45 s. **(A)** Subject stays still and eyes are being detected. Movement in the background to the right does not disrupt detection. **(B)** When the subject moves, several events are generated.

The indoor data set consists of recordings in controlled lighting conditions. Figure 5 shows tracking results. The algorithm starts tracking as soon as a single blink is detected (Figure 5A). Whereas tracking accuracy on the frame-based implementation is constant (25 fps), our algorithm is updated event-by-event depending on the movements in the scene. If the subject stays still, the amount of computation is drastically reduced as there is a significantly lower number of events. Head movement causes the tracker to update within μ*s* (Figure 5B).

Subjects in the outdoor experiments were asked to step from side to side in front of a camera placed in a courtyard under natural lighting conditions. They were asked to gaze into a general direction, partly engaged in a conversation with the person who recorded the video. Table 2 shows that results are similar to indoor conditions. The slight difference is due to the non-idealities of the sensor (same camera parameters as in the indoor experiment). It is important to emphasize that Event-based cameras still lack an automatic tuning system of their parameters that hopefully will be developed for the future generations of a cameras.

**Table 2:** Summary of results for detection and tracking for four sets of experiments.

	**No. of recordings**	**Blinks detected (%)**	**Error VJ (%)**	**Error FRCNN (%)**	**Error SSD (%)**	**Error CF (%)**
Indoor	21	68.4	5.92	9.42	9.21	10.51
Outdoor	21	52.3	7.6	14.57	15.08	14.88
Scale	3	62.6	4.8	10.17	10.22	17.6
Multiple	3	36.8	15	16.15	14.61	n/a
Total	48	59	7.68	11.77	11.52	12.82

*Percentage of blinks detected relates to the total number of blinks in a recording. Tracking errors are Euclidean distances in pixel between the proposed and respective method's bounding boxes, normalized by the frame-based bounding box width and height in order to account for different scales*.

### 3.2. Face Scale Changes

**Figure 6:**
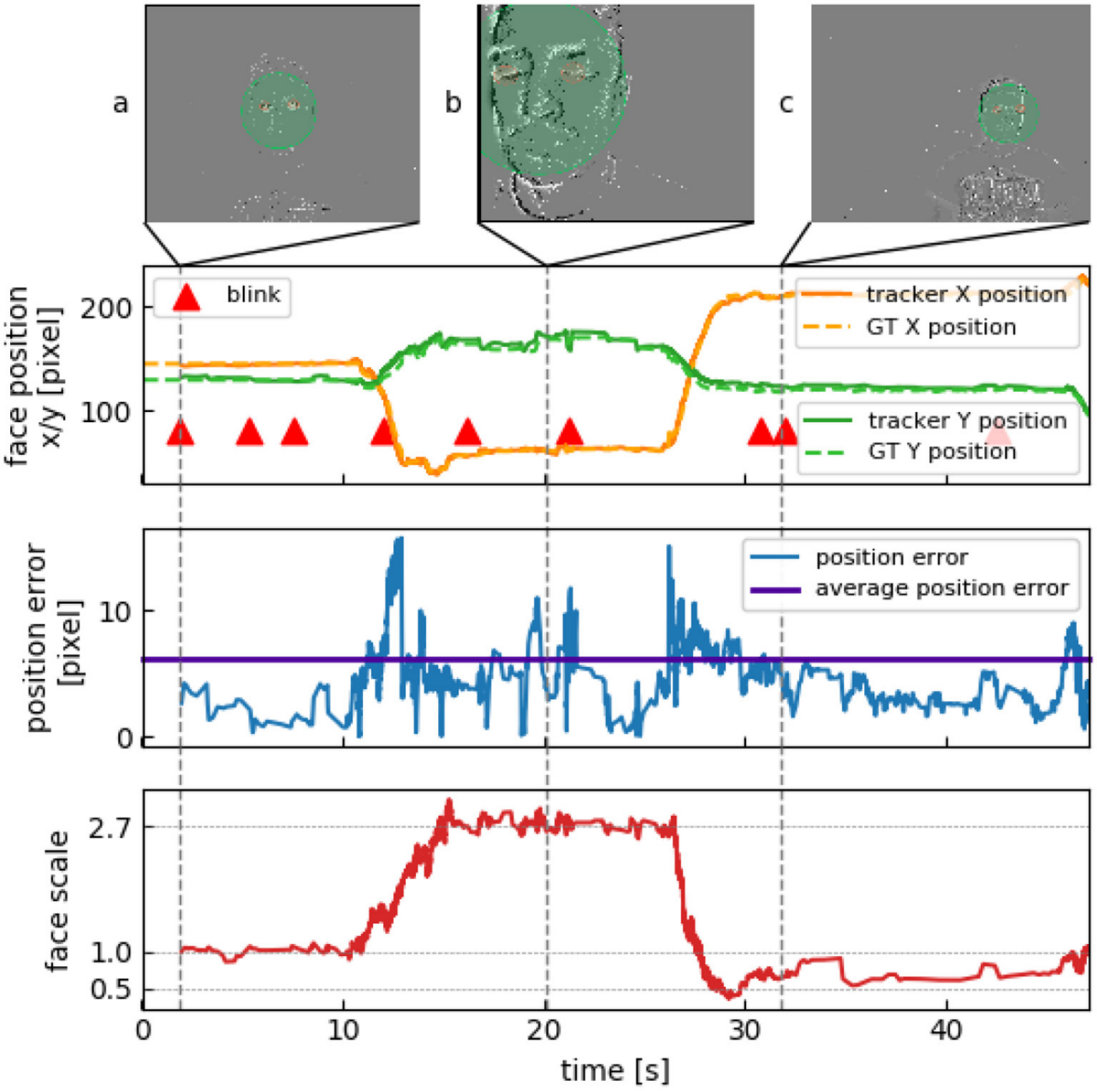
Verifying resistance to scale. **(A)** First blink is detected at initial location. Scale value of 1 is assigned. **(B)** Subject gets within 25 cm of the camera, resulting in a three-fold scale change. **(C)** Subject veers away to about 150 cm, the face is now 35% smaller than in **(A)**.

In three recordings the scale of a person's face varies by a factor of more than 5 between the smallest to the largest detected occurrence. Subjects were instructed to approach the camera within 25 cm from their initial position to then move away from the camera after 10 s to about 150 cm. Figure 6 shows tracking data for such a recording. The first blink is detected after 3 s at around 1 m in front of the camera (Figure 6A). The subject then moves very close to the camera and to the left so that not even the whole face bounding box is seen anymore (Figure 6B). Since the eyes are still visible, this is not a problem for the tracker. However, GT had to be partly manually annotated for this part of the recording, as two of the frame-based methods failed to detect the face that was too close to the camera. The subject then moves backwards and to the right, followed by further re-detections (Figure 6C).

### 3.3. Multiple Faces Detection

**Figure 7:**
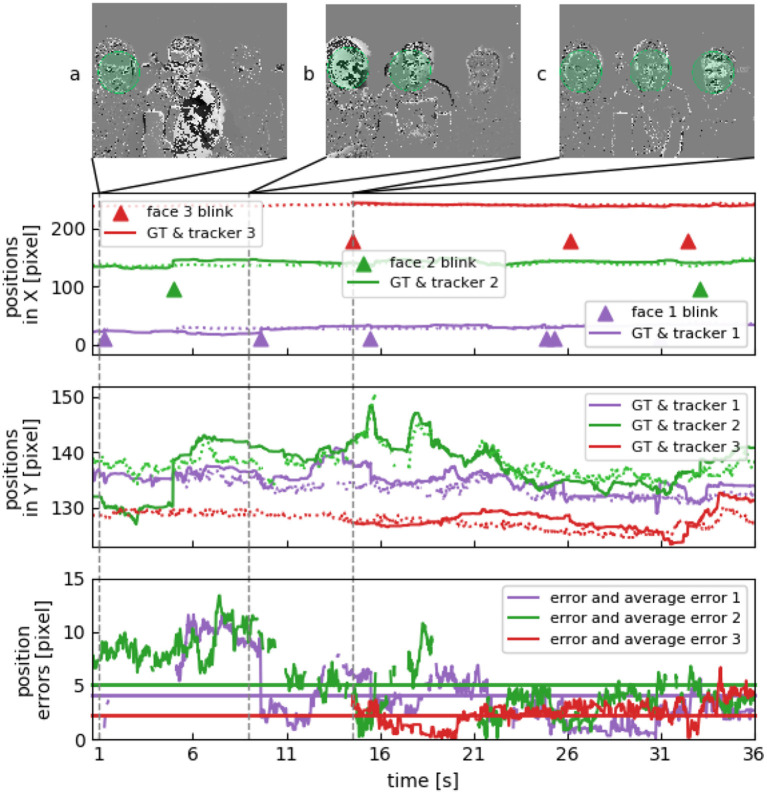
Multiple face tracking in parallel. Face positions in X and Y show three subjects sitting next to each other, their heads are roughly on the same height. **(A)** Subject to the left blinks at first. **(B)** Subject in the center blinks next, considerably varying their face orientation when looking at the other two. **(C)** Third subject stays relatively still.

We recorded three sets of three subjects sitting at a desk talking to each other. No instructions where given to the subjects. Figure 7 shows tracking results for the recording. The three subjects stay relatively still, but will look at each other from time to time as they are engaged in a conversation or sometimes focus on a screen in front of them. Lower detection rates (see Table 2) are caused by an increased pose variation, however this does not result in an increase of the tracking errors due to the absence of drift.

### 3.4. Pose Variation Sequences

**Figure 8:**
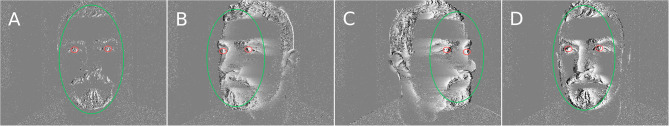
Pose variation experiment. **(A)** Face tracker is initialized after blink. **(B)** Subject turns to the left. **(C,D)** One eye is occluded, but tracker is able to recover.

The subjects in these sequences are rotating their head from one side to the other until only one eye is visible in the scene. Experiments show that the presence of a single eye does not affect the performances of the algorithm (see Figure 8). These experiments have been carried out with an event-based camera that has a VGA resolution. While this camera provides better temporal accuracy and spatial resolution, it does not provide gray-level events measurements.

Although we fed frames from the change detection events (which do not contain absolute gray-level information but are binary) to the frame-based methods, none of them could detect a face. This can be expected by the fact that the used networks have been trained on gray-level images. Perhaps if we re-train the last layers of the networks with manually labeled frames from change detection events (binary), they would probably achieve similar performances. However, the frame data set creation and the training are beyond the scope of this work.

### 3.5. Summary

Table 2 summarizes the relative accuracy of the detection and the tracking performances of the presented method, in comparison to VJ (Viola and Jones, 2004), FRCNN (Ren et al., 2015), SSD (Liu et al., 2016), and CF (Li and Shi, 2019). We set the correlation threshold to a value that is guaranteed to prohibit false positive detections, in order to (re-)initialize trackers at correct positions. The ratio of detected blinks is highest in controlled indoor conditions and detection rates in outdoor conditions are only slightly inferior. We attribute this fact to the aforementioned hardware limitations of earlier camera generations that are sensitive to lighting conditions. A lower detection rate for multiple subjects is mostly due to occluded blinks when subjects turn to speak to each other.

The tracking errors are the deviations from the frame-based bounding box center, normalized by the bounding box's width. The normalization provides a scale invariance so that errors estimated for a large bounding box from a close-up face have the same meaning as errors for a small bounding box of a face further away.

VJ, FRCNN, and SSD re-detect faces at every frame and discard face positions in previous frames, resulting in slightly erratic tracking over time. They do however give visually convincing results when it comes to accuracy, as they can detect a face right from the start of the recording and at greater pose variation given the complex model of a neural network. CF uses a tracker that updates its position at every frame that is created from binning the change detection events, rather than working on gray-level frames. The tracker update at each frame based on the previous position ensures a certain spatial consistency and smoothness when tracking, at the temporal resolution of the frame rate. However, since a correlation filter was initially designed for classic (gray-level) images, it relies on visual information of the object to track to be present at all time, which is not necessarily the case for an event-camera.

The CF technique from Li and Shi (2019) requires the camera to move constantly in order to obtain visual information from the scene to maintain the tracking, as the algorithms uses rate-coded frames. This required us to modify their algorithm since in our data, tracked subjects can stop w.r.t. to the camera, hence they became invisible. We added a mechanism to the correlation filter that freezes the tracker's position when the object disappears. We use a maximum threshold of the peak-to-sidelobe ratio (Bolme et al., 2010), which measures the strength of a correlation peak and can therefore be used to detect occlusions or tracking failure while being able to continue the online update when the subject reappears. This results in delays in tracking whenever an object starts to move again and results in tracking penalties. CF has further limitations at tracking at high scale variance and cannot track multiple objects of the same nature at the same time.

## 4. Conclusion

We introduced a method able to perform face detection and tracking using the output of an event-based camera. We have shown that these sensors can detect eye blinks in real time. This detection can then be used to initialize a tracker and avoid drifts. The approach makes use of dynamical properties of human faces rather than relying on an approach that only uses static information of faces and therefore only their spatial structure.

The face's location is updated at μs precision once the trackers have been initialized, which corresponds to the native temporal resolution of the camera. Tracking and re-detection are robust to more than a five-fold scale, corresponding to a distance in front of the camera ranging from 25 cm to 1.50 m. A blink provides robust temporal signatures as its overall duration changes little from subject to subject.

The amount of events received and therefore the resulting activity amplitude varies only substantially when lighting of the scene is extremely different (i.e., indoor office lighting vs bright outdoor sunlight). The model generated from an initial set of manually annotated blinks has proven to be robust to those changes across a wide set of sequences. The algorithm mechanism is also robust to eye occlusions and can still operate when face moves from side to side allowing only a single blink to be detected. In the most severe cases of occlusion, the tracker manages to reset correctly at the next detected blink.

The occlusion problem could be further mitigated by using additional trackers to track more facial features such as the mouth or the nose and by linking them to build a part-based model of a face as it has been tested successfully in Reverter Valeiras et al. (2015).

The blink detection approach is simple and yet robust enough for the technique to handle up to several faces simultaneously. We expect to be able to improve detection accuracy by learning the dynamics of blinks via techniques such as HOTS (Lagorce et al., 2016) or HATS (Sironi et al., 2018). At the same time with increasingly efficient event-based cameras providing higher spatial resolution, the algorithm is expected to increase its performance and range of operations. We roughly estimated the power consumption of the compared algorithms to provide numbers in terms of efficiency:

- The presented event-based algorithm runs in real-time using 70% of the resources of a single core of an Intel i5-7200U CPU for mobile Desktops, averaging to 5.5 W of power consumption while handling a temporal precision of 1μ*s* (Intel Corporation, 2017).
- The OpenCV implementation of VJ is able to operate at 24 of the 25 fps in real-time, using a full core at 7.5 W (Intel Corporation, 2017).
- The FRCNN Caffe implementation running on the GPU uses 175 W on average on a Nvidia Tesla K40c with 4–5 fps.
- The SSD implementation in Tensorflow runs in real-time, using 106 W on average on the same GPU model.

## Data Availability Statement

The face detection dataset for this study can be found under https://www.neuromorphic-vision.com/public/downloads/data-set-face-detection.tar.gz.

## Ethics Statement

Ethical review and approval was not required for the study on human participants in accordance with the local legislation and institutional requirements. Written informed consent to participate was not required in accordance with the national legislation and the institutional requirements. Written informed consent was obtained from the individuals for the publication of any potentially identifiable images or data included in this article.

## Author Contributions

GL, S-HI, and RB designed the algorithm. GL was responsible for data collection and programming. All authors participated in writing and editing the manuscript.

## Conflict of Interest

The authors declare that the research was conducted in the absence of any commercial or financial relationships that could be construed as a potential conflict of interest.

## Footnotes

^1^Dataset is available for download under https://www.neuromorphic-vision.com/public/downloads/data-set-face-detection.tar.gz; Supplementary Material.
